# Mapping the prevalence of household-scale livestock ownership by animal taxon in low- and middle-income countries: A prediction model using template model builder

**DOI:** 10.1371/journal.pone.0355207

**Published:** 2026-07-31

**Authors:** Josh M. Colston, Bin Fang, Vazira Ahmedjonova, Nasif Hossain, Prakrut Kansara, Francesca Schiaffino, Malena K. Nong, Adhvikaa Ambikapathi Revathi, Ben Zaitchik, Pavel Chernyavskiy, Venkataraman Lakshmi, Margaret N. Kosek

**Affiliations:** 1 Division of Infectious Disease and International Health, School of Medicine, University of Virginia, Charlottesville, Virginia, United States of America; 2 Department of Environmental Health and Engineering, The Johns Hopkins University, Baltimore, Maryland, United States of America; 3 College of Arts and Sciences, University of Virginia, Charlottesville, Virginia, United States of America; 4 Department of Statistics, Pabna University of Science and Technology, Pabna, Bangladesh; 5 The Environmental Institute, University of Virginia, Charlottesville, Virginia, United States of America; 6 Department of Earth and Planetary Sciences, Johns Hopkins University, Baltimore, Maryland, United States of America; 7 Faculty of Veterinary Medicine, Universidad Peruana Cayetano Heredia, Lima, Peru; 8 School of Medicine, University of Virginia, Charlottesville, Virginia, United States of America; 9 Department of Public Health Sciences, University of Virginia School of Medicine, Charlottesville, Virginia, United States of America; National Veterinary Research Institute (NVRI), NIGERIA

## Abstract

Animal husbandry is widely practiced on the household scale in communities in low- and middle-income countries (LMICs) and, while having economic and health benefits, exposes household members to risk of zoonotic infections to an extent that is unclear. While demand for georeferenced information on infectious disease risk factors and drivers is growing, spatial variation in livestock ownership remains poorly characterized at high resolution. This study aimed to use geostatistical methods to model and map the prevalence of livestock husbandry in LMICs for three major animal taxa: poultry, swine, and ruminants. Microdata relating to ownership of livestock animal species were sourced from various population-based survey programs which together cover the majority of LMICs and categorized. These were georeferenced and spatially matched with a panel of time-fixed environmental and demographic spatial covariates, INLA models were fitted to the resulting database, and probabilities for ownership of each livestock taxon predicted based on the model parameter estimates. The results indicated widespread poultry ownership across rural Central America, the Amazon basin, tropical Africa and river basins and forests of East Asia. Swine husbandry is the least widely practiced among the three livestock taxa and concentrated in an undulating belt of higher prevalence extending from central China, through southeast Asia to Northeastern India, though such predictions in data-sparse regions (particularly Muslim-majority areas) represent regional covariate patterns rather than fine-scale measurements. To address non-stationarity in swine spatial structure, region-specific spatial kernels were implemented. Rearing of ruminant livestock appears widespread across subequatorial Africa, Central Asia, the Gobi Desert, the Himalayas, Mongolia and northern India. The models perform impressively by most standard evaluation metrics, and the patterns in their predictions align with external evidence. The distribution of this important risk factor for infectious disease transmission can be modeled using publicly available data sources to generate plausible and potentially actionable predictions over wide geographic areas and identify regions of high exposure to animal disease reservoirs. The resulting predicted prevalence estimates are made available as supplementary files in GIS-compatible format.

## Introduction

Animal husbandry is widely practiced on the household scale in communities in low- and middle-income countries (LMICs) for its numerous economic and health benefits [1]. Around 70% of the world’s 880 million rural poor depend on traditional animal husbandry systems for their livelihoods (also referred to as “extensive livestock production”, as opposed to “intensive” or industrial-scale) [2]. In many low resource settings it is customary to rear livestock in close proximity to children, with poultry, swine and cattle sharing the domestic space and even the sleeping quarters with their owners’ household members [3,4]. For small-scale producers animals can be used as economic assets, to work in the fields or transport goods or for products such as eggs, milk, wool or meat which can be consumed by the family or sold on the market. When surveyed, many such farmers say that they enjoy living with animals, that their presence in the dwelling is important for their children’s education, and that home-reared animals are healthier and their food products tastier and more nutritious [5,6].

However, there is a trade-off between the benefits and risks of animal ownership. Domestic animals are also reservoirs for the many zoonotic pathogens that constitute emerging and endemic global health threats [7]. *Campylobacter*, for example, one of the agents responsible for the largest proportion of diarrheal disease morbidity in children in their second year of life, thrives in captive chicken populations and is frequently transmitted to humans from animal sources [8,9]. Other zoonotic pathogens of public health concern that may be transmitted from extensively farmed livestock to children include *E. coli*, salmonellosis, cryptosporidiosis, giardiasis [10,11], taeniasis/cysticercosis – contracted from pigs through ingestion of meat or via the fecal-oral route [12,13]; Japanese encephalitis – transmitted from pigs to humans via mosquitos [14]; and avian and swine influenza – transmitted respectively from poultry and pigs [15–17]. In fact, seventy five percent of emerging infectious diseases, and 61% of all human pathogens are capable of being transmitted from animals to humans [18], and the impacts to society that can result from the spillover of a zoonotic pathogen are starkly evident from the SARS-CoV-2 pandemic. Understanding how living in proximity to livestock impacts human health is a major research priority, and rural development initiatives increasingly emphasize sustainable livestock and waste management strategies [19]. Numerous largescale datasets exist for health and environmental indicators [20], however those resources that approximate the risk of contact between humans and animals have tended to focus on bushmeat hunting [21,22] or intensive rearing [23,24], and not the primary interface of human and domestic animal exposure, the household.

In this study from the Planetary Child Health & Enterics Observatory (Plan-EO, www.planeo.earth [25]) we aim to model and map the prevalence of livestock ownership in LMICs for three major animal taxa: poultry, swine, and ruminants. The guiding hypothesis is that the distribution and composition of animal husbandry, like other health-relevant exposures, varies systematically across ecological and demographic gradients in ways that can be captured through geostatistical modeling.

## Materials and methods

### Objective and scope

The objective of this study was to model the spatial variation in ownership of three major livestock taxa, and use the resulting estimates to map the predicted prevalence across LMIC’s (as defined by the Organisation for Economic Co-operation and Development [26], excluding those in Europe).

### Outcome variables

Both UNICEF’s Multiple Indicator Cluster Surveys (MICS) [27] and USAID’s Demographic and Health Surveys (DHS) [28] – which continues despite being terminated by the Trump administration in early 2025 [29] – contain household modules that include questions relating to ownership of assets, often (but not routinely) including each of a nationally tailored panel of livestock animal species. Several notable LMICs do not have recent MICS or DHS, instead implementing their own in-country surveys (e.g., Brazil, Nicaragua, Botswana etc.), however of these only the China Family Panel Studies (CFPS) [30] collected information about livestock ownership, and made them publicly available. Therefore, the outcome microdata for this study were sourced from these three population-based survey programs which together cover the majority of LMICs.

Specific livestock animal species for which there were questions in any survey were translated where necessary and classified into the broader poultry, swine, and ruminant taxa according to the schema in Table 1, to reflect biological taxonomy and common agricultural practices and classifications. We did not consider other monogastric livestock species, such as rodents or equids, since these were inconsistently included in surveys and not common to most geographies. All Standard DHSs, Malaria and AIDS Indicator Surveys (MIS and AIS), MICS and CFPS conducted since 2005 that included livestock ownership data from non-European LMICs were included. For countries lacking such surveys, comparable data from earlier surveys (2000–2004) were used where available. While many surveys ask for the total number or head of each animal owned, several large surveys (notably the 2006, 2016, and 2021 India DHSs) instead phrased the questions as a binary yes/no choice. For consistency and inclusiveness, we therefore reclassified the livestock information from all surveys into binary ownership/non-ownership categories. Some surveys of majority Muslim countries did not include questions about swine ownership in any surveys that asked about other livestock species, while others asked in some surveys but not others. In the former case, it was assumed that ownership of pigs and swine was negligible for religious and cultural reasons, and households with information on other livestock were coded as “no” for swine. In the latter, because swine ownership was significant enough to have been included in some surveys, for those from which it was excluded, all households were coded as having missing values for swine ownership. A sensitivity analysis comparing this decision to treating all households in surveys that did not ask about swine ownership as missing found the results to be robust.

**Table 1:** Classification of livestock into three major animal taxa.

Animal Taxon	Livestock
Poultry	Chickens, ducks, geese, turkeys, swans, quails, guineafowl, pigeons, cassowary
Swine	Pigs, boar
Ruminants^*^	Cattle, buffalos, oxen, bulls, yaks/naks, zebu, sheep, goats, camels

*Including pseudo-ruminant camelids

### Covariates

A panel of time-fixed environmental and demographic spatial covariates were chosen based on their hypothesized associations with the outcome variables, compiled in raster format, and their values extracted at the georeferenced locations of the primary sampling units (sampled neighborhoods or communities, known as “clusters”) using Python. Definitions and sources of each covariate are shown in Table 2. In addition, time was calculated in continuous months since January 1^st^, 2005, based on the date of survey interview and log-transformed.

**Table 2:** Definitions and sources of variables included as covariate predictors in the model.

Variable	Definition	Units/Categories^1^	Source
Accessibility to cities	Travel time to nearest settlement of >50,000 inhabitants.	Minutes	MAP [31]
Aridity index	Mean annual precipitation / Mean annual reference evapotranspiration, 1970–2000.	Ratio	CGIAR-CSI [32]
Climate zone	First level Köppen-Geiger climate classification.	Tropical; arid; temperate; cold; polar	Beck et al. 2018 [33]
Cropland areas	Proportion of land given over to cropland, 2000.	Proportion	CIESIN [34]
Distance to major river	Distance to major perennial river (derived from rivers and lakes centerlines database).	Decimal degrees	Natural Earth [35]
Elevation	Elevation above sea level.	Meters	NOAA [36]
Economic development	Sub-national unit-level Gross Domestic Production (GDP) per capita, 2015	Constant 2011 int. USD	Kummu et al. 2018 [37]
Enhanced Vegetation Index	Vegetation greenness corrected for atmospheric conditions and canopy background noise.	Ratio	USGS [38]
Growing season length	Reference length of annual agricultural growing period (baseline period 1961–1990).	Days	FAO, IIASA [39]
Human development	Sub-national unit-level Human Development Index (HDI), 2015	Scale from 0 to 1	Kummu et al. 2018 [37]
Human Footprint Index	Human Influence Index (HII) normalized by biome and realm.	Percentage	CIESIN [40]
Income inequality	Gini coefficient based on disposable income	Scale from 0 to 1	Chrisendo et al. 2025 [41]
Irrigated areas	Percentage of land equipped for irrigation around the year, 2000.	Percentage	FAO [42]
Land cover and use	General class of vegetation, tree, and ice cover or purpose of land use, 2020 (resampled and reclassified from Global Land Cover and Land Use)	Built up; cropland; desert; semi-arid; short vegetation; snow or ice; tree cover; wetland	GLAD [43]
Land Surface Temperature	Interannual averages of daily land surface temperature estimates for daytime, nighttime, and day/nighttime range, 2003–2020.	K	MOD21A1N v006 [44,45]
Nighttime light	The surface upward radiance from artificial light emissions extracted from at-sensor nighttime radiances at top-of-atmosphere.	nWatts·cm ^− 2^·sr ^− 1^	NASA Black Marble [46]
Pasture areas	Proportion of land given over to pasture, 2000.	Proportion	CIESIN [34]
Population density	Human population density per 1km^2^.	Inhabitants per km^2^	WorldPop [47]
Potential evapotranspiration	8-day sum of the water vapor flux under ideal conditions of complete ground cover by plants.	kg/m²/8-day	NASA EOSDIS [48]
Region*	Region of the globe as defined by the World Bank	East Asia & Pacific; Europe & Central Asia; Latin America & the Caribbean; Middle East & North Africa; South Asia	World Bank [49]
Soil type	Texture or classification of soil used to predict water holding capacity.	Coarse; Medium; Medium fine; Fine; Very fine; Organic; Tropical organic.	ECMWF Integrated Forecast System [50,51]
Soil moisture	Water content of soil	m^3^/m^3^	Fang et al. 2025 [52]
Urbanicity	Urbanicity status at georeferenced location (reclassified from Global Human Settlement database).	Urban; peri-urban; rural; remote	GHS [53]

* Only included in the swine model.

### Statistical analysis

Households were georeferenced using an automated algorithmic cluster location assignment process that has been documented previously [20]. Spatial autocorrelation was explored by generating semi-variograms of Pearson residuals from non-spatial logistic regression models to determine the appropriate spatial covariance function. Three spatially explicit logistic models were fitted, one to each of the binary outcome variables using the R Template Model Builder (RTMB) package in which locations are projected onto a coarsened mesh of vertices that carry the spatial information and can be reprojected onto the observed data [54,55]. All coordinates were transformed via the Mollweide projection and scaled into kilometers prior to analysis. The meshes used for modeling poultry and ruminants had 29,926 vertices, while the swine mesh had 29,580. For poultry and ruminants, the semivariograms indicated that the spatial correlation could be captured using a stationary Matérn covariance function, while for swine, an irregular pattern of semi-variance necessitated the use of a non-stationary, region-varying Matérn structure (S1 Fig in S1 File). We therefore fitted region-specific Matérn kernels for swine by including region variable as fixed effects, while poultry and ruminants retained global SPDE specifications. This region-stratified approach substantially improved model fit for swine, particularly in capturing the cultural and economic heterogeneity in swine practices across regions. The predictive performance of the spatial models was assessed using leave-one-country-out (LOCO) spatial cross-validation whereby all observations from a single country are excluded, the models are refitted to the remaining data, predictions are made for left-out households, and performance metrics (recall, precision, accuracy, F1-score, and area under the receiver operating characteristic curve (ROC-AUC) statistics) are computed per fold and averaged. To manage computational cost, we evaluated a stratified random subsample of 20 countries balanced across region variable and livestock components. To assess covariate collinearity, variance-inflation factors (VIF) were computed on the fitted logistic model for each taxon (Supplementary Table S4 in S1 File). Development-related covariates (nighttime lights, built-up footprint, population density) exhibit moderate collinearity (VIF = 3–8), reflecting spatial co-occurrence in urbanized areas. This collinearity does not bias coefficient estimates or predictions. Contributions of each covariate to the models were quantified, assessed and visualized by calculating and plotting their Shapley Additive Explanation (SHAP) values [56].

### Model predictions

Predicted probabilities for each outcome were made for all locations in the domain of interest at 0.05 decimal degree resolution (~5.6 km at the equator) and exported in Georeferenced Tag Image File (GeoTIFF) format based on a polygon boundary shapefile for all LMICs obtained from the U.S. Department of State—Humanitarian Information Unit (available in the public domain with no restrictions, CC0) [57]. The spatial covariates from Table 2 along with the time variable were used to generate predicted logistic distribution probabilities of a household reporting the presence of animals of that taxon from the corresponding model. A value for time corresponding to the first of January 2023 was used for making predictions. Input raster covariates were assessed for missing pixels across the prediction domain. Per-covariate missingness is reported in Supplementary Table S5 in S1 File. Most covariates have <1% missing pixels on land (range 0–0.3%). Missing pixel values were filled by performing imputation using k-Nearest Neighbors method (k = 10) by Python Scikit-learn package [58].

### Ethics statement

All human subject information used in this analysis was anonymized, publicly available secondary microdata, and therefore ethical approval was not required or sought. For data provided by the DHS Program, data access requests (including for the displaced cluster coordinates) were submitted and authorized and data downloaded through the Program’s website. Survey microdata was stored and analyzed in a secure, virtual computing environment. A completed checklist of Guidelines for Accurate and Transparent Health Estimates Reporting (GATHER [59]) is included in Supplementary File S1 in S1 File.

## Results

**Fig 1:**
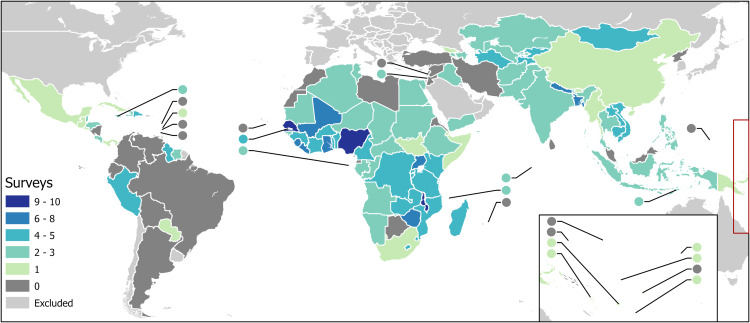
Number of nationally representative household surveys included in input dataset by country for included LMICs. Small countries are represented by circles. Base map compiled from shapefiles obtained from U.S. Department of State—Humanitarian Information Unit (https://data.humdata.org/dataset/global-lsib-polygons-detailed) and Natural Earth free vector map data (https://www.naturalearthdata.com) that are made available in the public domain with no restrictions (CC0).

Out of 378 surveys otherwise eligible for inclusion in this study, 306 (81.0%) provided data relating to ownership of animals in the three target taxa in a total of 5.3 million households in 241,000 clusters across 96 countries (Fig 1). S2 File gives the national level distribution of each of the three livestock taxon variables in each survey (without sample weights applied). All eligible surveys included information on poultry ownership however, two of these (conducted in the island nations of Kiribati and Tuvalu) had not asked about any animals in the ruminant taxon and 38 had not included questions on swine ownership, mostly in predominantly Muslim countries, though not exclusively (e.g., Cambodia, Guyana, Haiti). Numerous notable LMICs either did not have any surveys available in the public domain (e.g., Iran, Libya, Eritrea) or had surveys that did not ask about livestock ownership, which was especially the case for most South American countries. S1 Fig in S1 File shows Pearson residual semi-variograms for each variable. While poultry and ruminants exhibited spatial autocorrelation patterns that can be adequately captured by a Matern assumption, the irregular pattern for swine was suggestive of a non-stationary random field that may violate the stationarity assumption.

**Fig 2:**
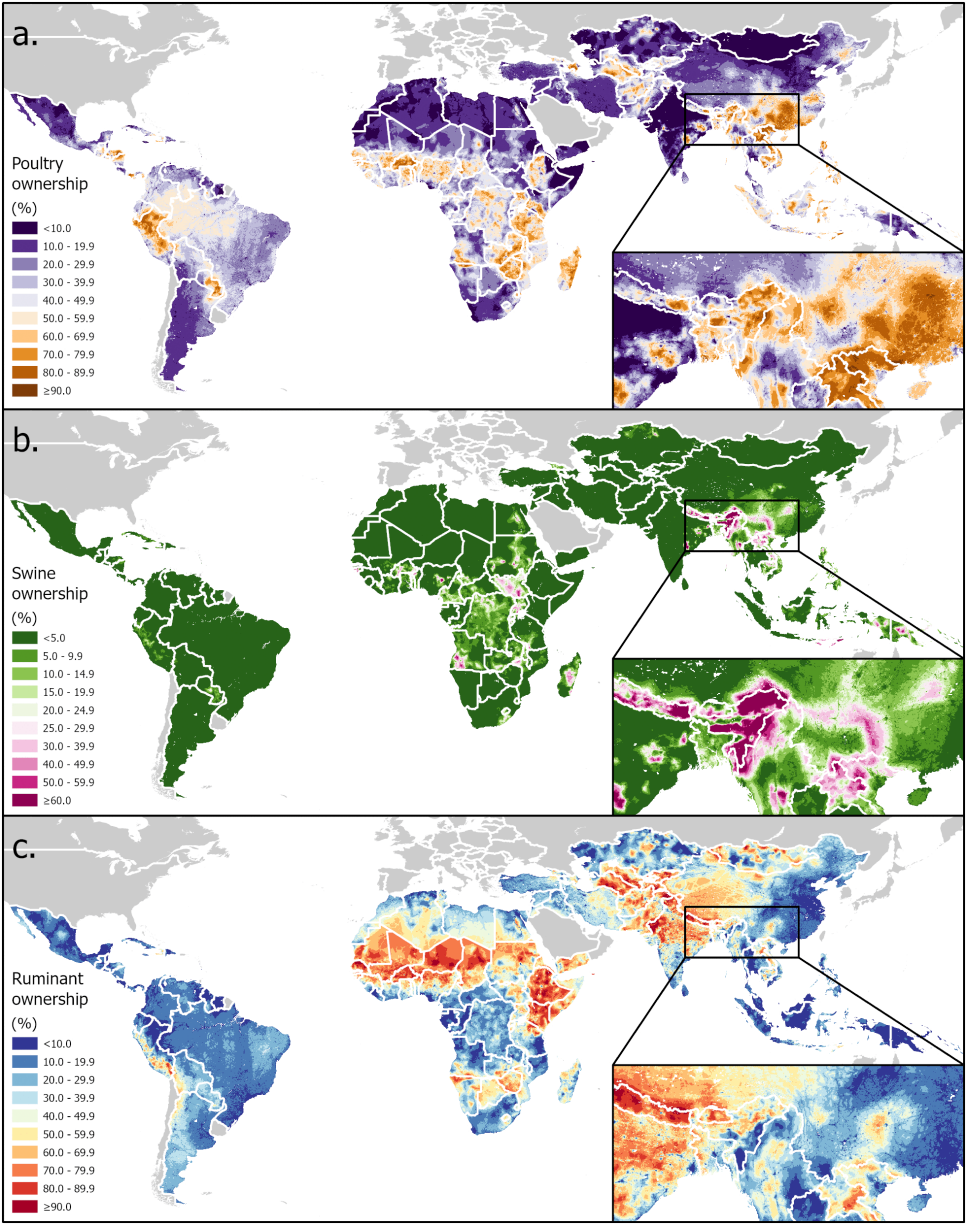
Prevalence of ownership for three livestock animal taxa – a. poultry, b. swine, c. ruminants – in LMICs predicted by models fitted to household survey data in RTMB. Inset maps show details at a smaller zoom extent. Base maps compiled from shapefiles obtained from U.S. Department of State—Humanitarian Information Unit (https://data.humdata.org/dataset/global-lsib-polygons-detailed) and Natural Earth free vector map data (https://www.naturalearthdata.com) that are made available in the public domain with no restrictions (CC0).

Fig 2 shows the subnational geographical distribution of the prevalence of livestock ownership predicted by the models for each of the three animal taxa (predictions that are also provided as supplementary GeoTIFF raster files available on the Dryad data repository at this URL https://doi.org/10.5061/dryad.n2z34tnb3). In the Americas, poultry ownership appears most widespread across large rural areas of Central America (with the exception of Costa Rica), the interior of Peru, and central and southern Paraguay, though smaller pockets occur on Haiti’s Tiburon Peninsula, Cuba’s eastern extreme, and Guyana’s interior. In Africa, poultry ownership is concentrated in patches along the Tropical Savanna regions, around the East African Great Lakes and Rift Mountains, in parts of the Congo Basin, Angola and its border with Namibia, and across much of Madagascar, while remaining low across Africa’s far north and south and the Somali Peninsula. The largest focus of poultry rearing in Asia spans the Mekong, Pearl, and Red River basins and subtropical evergreen forests of Southern China, Northern Vietnam, and Loas, stretching into Myanmar, Cambodia, and northeast India. Other pockets occur in Turkmenistan, Azerbaijan, and the Malay Peninsula including most of the Sulawesi, and Lesser Sunda Islands (including Timor-Leste), parts of Indonesian Borneo, Sumatra and Java, and areas of the Philippines’ Luzon and Mindanao. Poultry rearing is low in the Middle East and largely absent from Mongolia and most of India, except for some pockets in the country’s east.

Swine husbandry is the least widely practiced among the three livestock taxa, being virtually non-existent in Latin America, West and Central Asia, North Africa and the Sahel, Mongolia and Northern China, and most of India and Indonesia. In continental Asia, a belt of higher prevalence extends across Nepal and Northeastern India and neighboring areas of Myanmar, China, and Bhutan, with other pockets occurring in southeast India, and China and northern Vietnam and Laos. The few other areas where the model predicts the proportion of households practicing swine husbandry to exceed 50% include the New Guinea Highlands, the Lesser Sunda Islands, and small pockets in Africa (central Madagascar and Nigeria, coastal Guinea-Bissau, southwestern Angola, and southern South Sudan). Rearing of ruminant livestock has a less focal predicted distribution than the other two taxa, appearing to be widespread across the Sahara, Sahel, Sudanian Savanna and Horn of Africa, as well as Central Asia, the Gobi Desert, the Himalayas, Mongolia and northern India. In the Americas the practice is less common, being mainly confined to pockets in the Andes, southern Paraguay, and Haiti.

**Fig 3:**
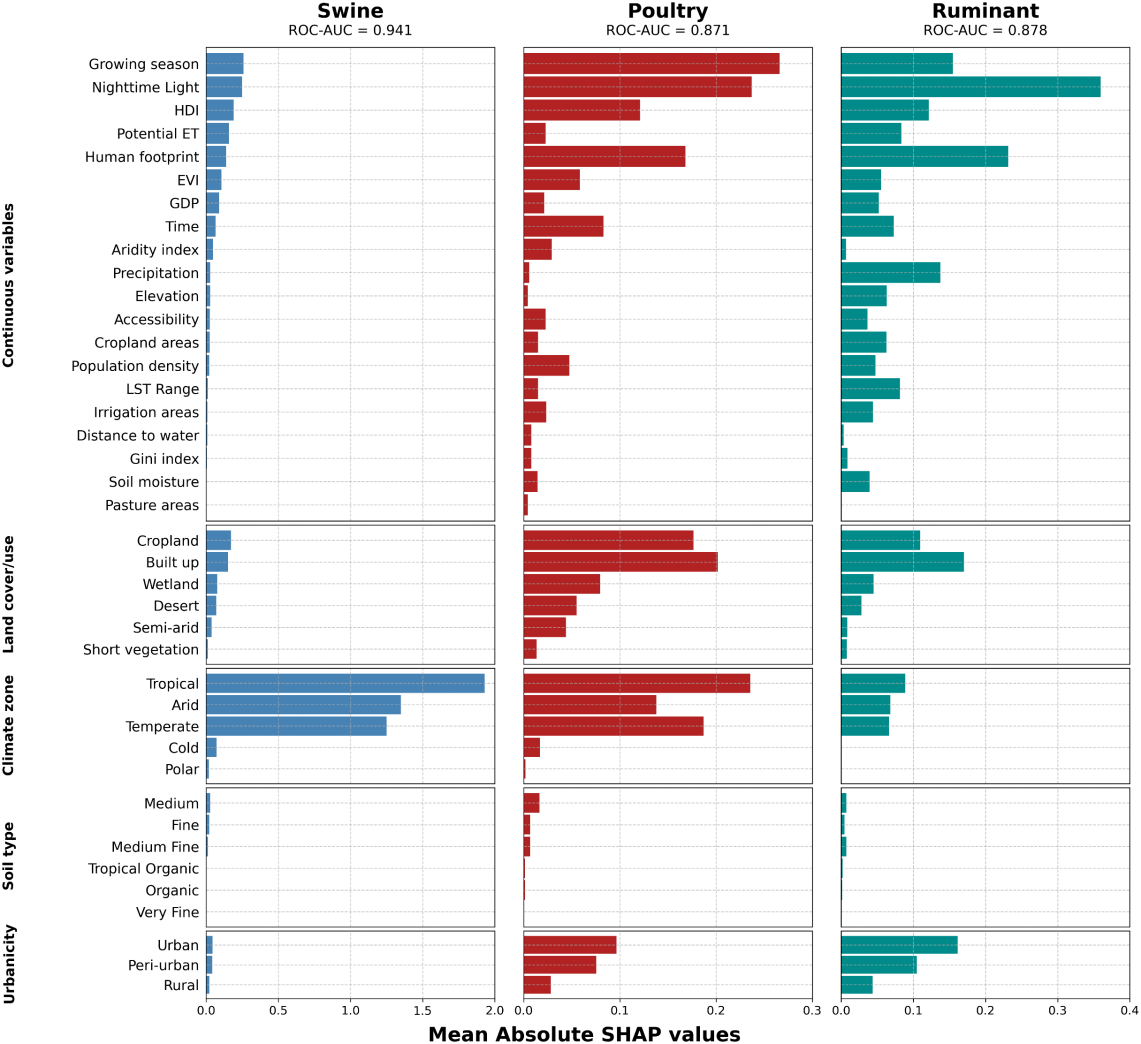
Feature importance for each of the variables and their categories included in the final model for each of the livestock animal taxa. HDI – Human Development Index; EVI – Enhanced Vegetation Index; LST – Land Surface Temperature; ET – Evapotranspiration; GDP – Gross Domestic Product; MENA – Middle East and North Africa; LAC – Latin America and the Caribbean; SA – South Asia; EAP – East Asia and Pacific; SSA – Sub-Saharan Africa. Comparison categories for factor variables are Land cover/use – tree cover; Climate zone – tropical; Region – Europe and Central Asia; Urbanicity – remote.

Fig 3 reports the SHAP values for each covariate (and value of the factor variables) as an approximation of their contribution to, along with the overall ROC-AUC statistics for each of the three models. Other model performance statistics, including the results of the LOCO cross-validation are reported in supplementary Tables S1 and S2 in S1 File. The ROC-AUC values for all models were suggestive of a high ability to discriminate between livestock owning and non-owning households, with the swine model performing particularly promisingly on this metric. For all three livestock taxon models, climate zone was an influential variable, particularly tropical areas for poultry and for swine, as were the “built up” and “cropland” land use categories. Among the other variables, growing season length and nighttime lights had a high impact on all three models’ predictions, while income inequality, distance to water, and pasture areas had low impact.

## Discussion

Daily contact with animals is a fact of life for young children growing up in rural communities in the less developed regions of the world and can be a significant source of infections through exposure to fecal contamination and respiratory pathogens [6,62]. As child health challenges in LMICs become increasingly localized, there is an urgent need for spatially resolved data on exposures that influence the risk of infectious disease transmission. Despite the well-recognized importance of household livestock in remote and rural livelihoods and its potential role in zoonotic pathogen transmission, spatial variation in livestock ownership remains poorly characterized at high resolution. However, demand for georeferenced information on livestock ownership patterns is likely to grow, as community-based development programs increasingly focus on sustainable animal waste management strategies [60]. To address this gap, this analysis modeled the small-scale spatial variation in livestock ownership prevalence using covariates with quasi-global coverage and used the results to map the predicted prevalence of these practices, estimates that we make freely available to researchers in a widely used GIS-compatible format for use in their own analyses.

While the models perform impressively by most standard evaluation metrics, perhaps more encouraging is the extent to which the patterns in their predictions align with external evidence. Results from the swine ownership model are particularly illustrative of this. An especially well delineated focus of smallholder swine rearing is predicted in the highlands of Papua New Guinea, a region where pigs are known to be prized agricultural assets, well adapted to the high altitude environment [61]. On the Indonesian side of the island of New Guinea, which is similarly mountainous, but with a much higher proportion of the population practicing Islam, lower prevalence of swine ownership is predicted. In fact, the one province of Indonesia where a high prevalence of this variable was predicted was in the majority Christian East Nusa Tenggara (along with the neighboring Christian nation of Timor-Leste) where smallholder commercial pig production has been incentivized through development programs for its socio-cultural acceptability and economic benefits [62,63]. The strong influence of religion constraining swine ownership is also evident in Guinea Bissau, where a high concentration was predicted in the coastal regions, where Christianity and traditional African religions are practiced, contrasting with the virtual absence of ownership in the majority Muslim interior of the country and neighboring Guinea and Senegal [64].Pig rearing in India is disproportionately practiced by disadvantaged communities and concentrated in the hilly regions of the country’s northeast (home to 46% of the national pig population) [65], a geographical distribution which the model successfully captures along with other pockets of swine rearing in Asia such as northern Laos [66] and the Tibetan plateau [67].

Similarly explicable patterns emerge for the other livestock taxa. The broad belt of high ruminant ownership spanning the continent of Africa across the subequatorial latitudes and arcing southwards to the horn of Africa, reflects the relative abundance of rangelands and pasture that have historically supported the flourishing of pastoralist, often nomadic societies and the overlapping rearing of cattle, sheep and goats [68–70]. National surveys in countries across this belt of Africa (as well as those in central and south Asia) typically ask respondents about camels when enumerating livestock species [28]. While camelids are not classified taxonomically as true ruminants they are often thought of as pseudo-ruminants due to similarities in anatomy, feeding behaviors, husbandry practices and disease susceptibility between the two taxa [71,72]. We chose to include group camels together with ruminants (no surveys asked about llama or alpaca ownership) for these reasons, and particularly because of their documented role in zoonotic spillovers to humans when kept as livestock, notably the Middle East Respiratory Syndrome Coronavirus (MERS-CoV) [73]. Several areas of deserts, plains and savannas that exhibit high prevalence of ruminant ownership have correspondingly low levels of poultry rearing, such as the whole of Mongolia, north, central and western India, and the Horn of Africa, perhaps because seasonal temperature and rainfall exchange render poultry vulnerable to heat stress and water scarcity [74].

There are several limitations to this study. Our classification of livestock species into broad taxa was informed by transmission mechanisms of enteric pathogens (the primary focus of the Plan-EO initiative), but may be too course to address questions related to pathogens for which certain ruminant or poultry species are the sole or primary reservoir, as cattle are for Bovine Tuberculosis and Brucellosis [75]. Furthermore, we treated animal ownership as a binary variable due to inconsistencies in the way the source data was coded, however there may be some hypotheses for which density of animals may be more important than prevalence. Geographical coverage of our outcome variables was uneven and sparse in many areas, especially South America, due both to lack of survey data for some LMICs, or the exclusion of livestock related questions from surveys in others. This meant that we had to extrapolate our predictions based on spatial variables to many large countries such as Brazil, Argentina, Iran and Libya, thus increasing their uncertainty for those countries. Interpretation of the predictions for these data-poor regions should take into account their increased uncertainty, as quantified by the standard errors for the predictions, which are provided as supplementary files. Finally, our analysis focused on what we consider the primary animal-human interface from the point of view of zoonotic disease transmission – namely the peridomestic environment of smallholder livestock farmers – but does not consider others which may be growing importance such as wet market trading and bushmeat hunting.

In conclusion, and these limitations notwithstanding, the distribution of this important risk factor for infectious disease transmission can be modeled using publicly available data sources to generate plausible and potentially actionable predictions over wide geographic areas and provide geographically resolved proxy exposure layers that may support future zoonotic disease risk stratification when combined with other epidemiological or environmental data.

## Supporting information

S1 File Supplementary methods, results, and guideline compliance. (DOCX)

S2 File National level distribution of each of the three livestock animal taxa in each contributing survey. (XLSX)

S3 File Spatial variation in livestock ownership in low- and middle-income countries – raster files of prevalence estimates and standard errors. (ZIP)
