# Supplementary material for: Mapping the prevalence of household-scale livestock ownership by animal taxon in low- and middle-income countries: A prediction model using template model builder
Source: PLoS One. 2026 Jul 31;21(7):e0355207. doi: 10.1371/journal.pone.0355207 (PMC13426965; doi:10.1371/journal.pone.0355207)
Supplement: S1 File — (DOCX) [file pone.0355207.s001.docx]

**Supplementary Methods, Results, and Guideline Compliance:**

Mapping the prevalence of household-scale livestock ownership by animal taxon in low- and middle-income countries: a RTMB prediction model

## ***Exploratory variography***

Semi-variograms of Pearson residuals from non-spatial logistic regression models were constructed using the gstat package, as shown in **Figure S1**.


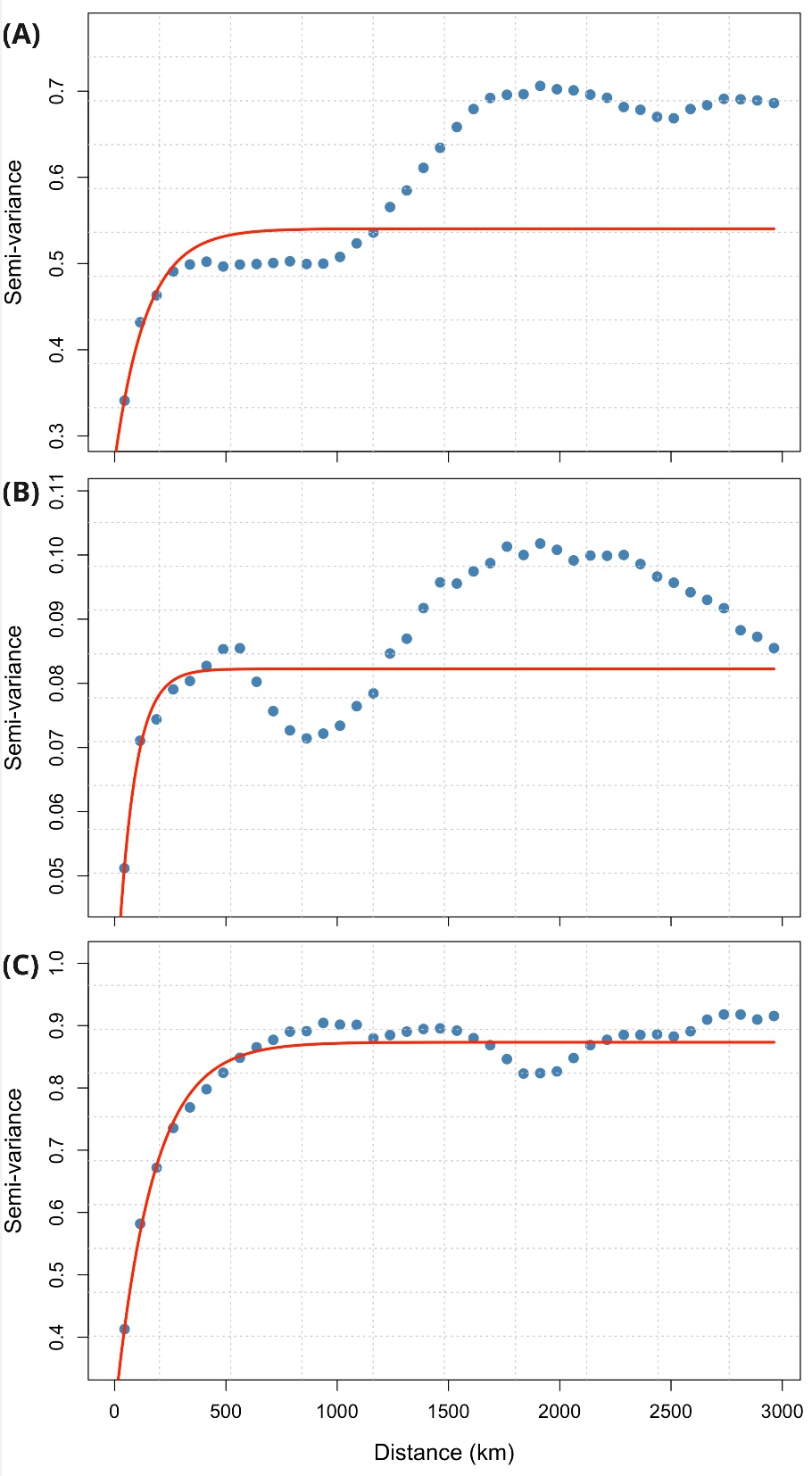


**Figure S1:** Pearson residual semi-variograms for prevalence of ownership of poultry **(A)**, swine **(B)**, and ruminants **(C)**. Coordinates were projected via the Mollweide projection and rescaled to kilometers prior to analysis.

## ***SPDE mesh construction***

The SPDE “mesh” was constructed on projected coordinates with continental borders treated as a boundary (**Figure S2A**). The maximum edge length defines the precision of the mesh: smaller edges produce more refined meshes. However, we must balance this precision with the associated computational burden. Here, we took the maximum edge to be 155.73km, which is less than the estimated range of the spatial field (from the variograms). The resultant total number of vertices ranges 29,580-29,926. Household locations overlaid over the mesh are shown in **Figure S2B**.


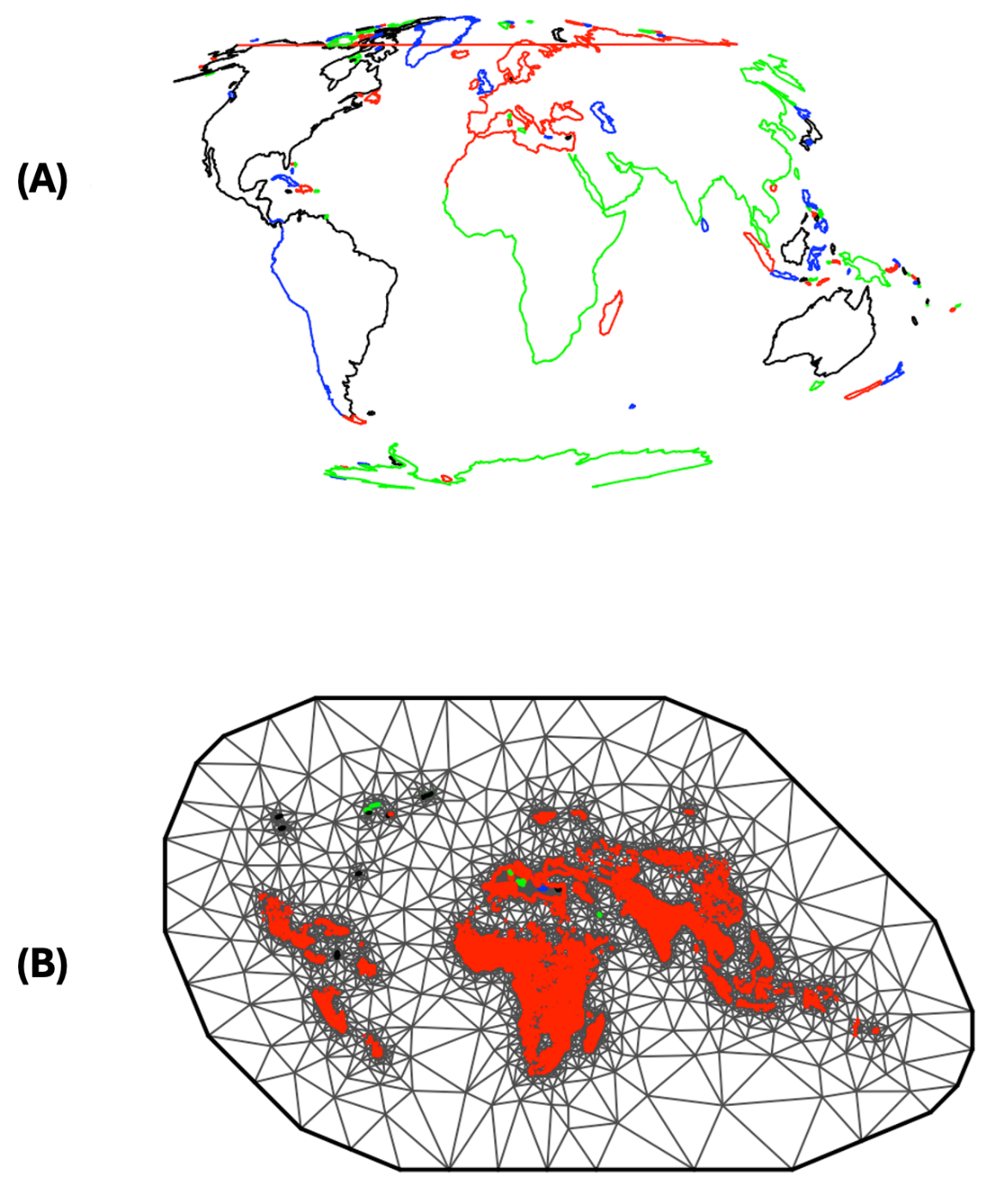


**Figure S2:** Continental borders used as the boundary for SPDE mesh construction based on polygon shapefiles obtained from U.S. Department of State—Humanitarian Information Unit (https://data.humdata.org/dataset/global-lsib-polygons-detailed) and made available in the public domain with no restrictions (CC0). Coordinates and borders were projected via the Mollweide projection prior to analysis shapefiles **(A)**. SPDE mesh with 29,580 vertices and locations of households included into the model (red points) **(B)**.

## ***RTMB statistical model and priors used***

All outcome variables in this study were binary; thus, the likelihoods used were Bernoulli with probability ${\pi(\boldsymbol{s}}_{i})$, which necessarily varies with location $\boldsymbol{s}_{i}$. The RTMB latent Gaussian Random Field (GF) models for each outcome were specified as follows:

$$Y\left( \boldsymbol{s}_{i} \right)|u\left( \boldsymbol{s}_{i} \right)\sim Bernoulli({\pi(\boldsymbol{s}}_{i}))$$

$$logit\left( {\pi(\boldsymbol{s}}_{i} \right)|u\left( \boldsymbol{s}_{i} \right))=\alpha+x^{T}\left( \boldsymbol{s}_{i} \right)\boldsymbol{\beta}+u\left( \boldsymbol{s}_{i} \right)$$

$u\left( \boldsymbol{s}_{1},\ldots,\boldsymbol{s}_{N} \right)\sim GF\left[ \boldsymbol{0}, \boldsymbol{\Sigma}_{Matérn} \right]$,

where $u\left( \boldsymbol{s}_{i} \right)$ is the GF evaluated at location $\boldsymbol{s}_{i}$, $\alpha$ is the intercept, and $x^{T}\left( \boldsymbol{s}_{i} \right)$ consists of all fixed effects shown in Table 2 recorded at location $\boldsymbol{s}_{i}$. The stationary Matérn covariance function ${\boldsymbol{(\Sigma}_{Matérn}\boldsymbol{)}}_{ij}=COV\left( u\left( \boldsymbol{s}_{i} \right),u\left( \boldsymbol{s}_{j} \right) \right)$ is parameterized as follows:

$COV\left( u\left( \boldsymbol{s}_{i} \right),u\left( \boldsymbol{s}_{j} \right) \right)=\frac{\sigma_{u}^{2}}{2^{1-\nu}\Gamma\left( \nu\right)}\left( \kappa h \right)^{\nu}K_{\nu}\left( \kappa h \right)$,

where $h=\left\| \boldsymbol{s}_{i}-\boldsymbol{s}_{j} \right\|$, $\sigma_{u}^{2}$ is the variance of the GF, $\kappa$ is a scale parameter, $\nu$ is a smoothness parameter, and $K_{\nu}$ is the modified Bessel function of the second kind of order $\nu$. Cameletti and colleagues showed that the practical range $\rho=\sqrt{8\nu}/\kappa$, which is estimated and reported by RTMB, is the distance at which between-location correlation is approximately 0.1 (1).

RTMB requires prior distributions be assigned to all hyperparameters of the GF. In our case, the log-hyperparameters consist of the standard deviation $\sigma_{u}$ and (effective) range parameter $\rho$. We regularized the log-hyperparameters with weak Gaussian priors, and the spatial field ω and cluster intercepts vc were treated as random effects and integrated out via the Laplace approximation implemented in RTMB, and the remaining parameters were estimated by penalized maximum likelihood (nlminb). For our data, we specified:

$\log\left( \tau\right)\sim Normal\left( 0,1 \right)\log\left( \kappa\right)\sim Normal\left( 0,1 \right)\log\left( \sigma_{u} \right)\sim Normal\left( 0,1 \right)$.

## ***Supplementary results***

| **Table S1**: Evaluation statistics for the three models of livestock ownership | | | | | | |
| --- | --- | --- | --- | --- | --- | --- |
|  | | **Observations (%)** | **Precision** | **Recall** | **F1-score** | **ROC-AUC** |
| **Poultry** | **Total** | 4,986,083 (100) | - | - | - | 0.871 |
|  | **No** | 3,367,999 (67.5) | 0.9 | 0.75 | 0.82 | - |
|  | **Yes** | 1,618,084 (32.5) | 0.61 | 0.83 | 0.7 | - |
|  | **Macro-average** | - | 0.75 | 0.79 | 0.76 | - |
|  | **Weighted average** | - | 0.81 | 0.77 | 0.78 | - |
| **Swine** | **Total** | 3,812,889 (100) |  |  |  | 0.941 |
|  | **No** | 3,516,349 (92.2) | 0.99 | 0.83 | 0.9 | - |
|  | **Yes** | 296,540 (7.8) | 0.31 | 0.91 | 0.46 | - |
|  | **Macro-average** | - | 0.65 | 0.87 | 0.68 | - |
|  | **Weighted average** | - | 0.94 | 0.83 | 0.87 | - |
| **Ruminants** | **Total** | 4,986,083 (100) |  |  |  | 0.878 |
|  | **No** | 3,232,009 (64.8) | 0.89 | 0.76 | 0.82 | - |
|  | **Yes** | 1,754,074 (35.2) | 0.65 | 0.83 | 0.73 | - |
|  | **Macro-average** | - | 0.77 | 0.79 | 0.77 | - |
|  | **Weighted average** | - | 0.81 | 0.78 | 0.79 | - |

| Table S2: Leave-One-Country-Out (LOCO) Cross-Validation Performance Metrics | | | | | | | | | | | |
| --- | --- | --- | --- | --- | --- | --- | --- | --- | --- | --- | --- |
| Model | Evaluation | AUC | | Recall | | F1 | | Accuracy | | n_pos | |
| Poultry | LOCO:HTI | 0.648 | | 0.794 | | 0.686 | | 0.629 | | 15871 | |
|  | LOCO:NER | 0.561 | | 0.905 | | 0.595 | | 0.505 | | 6176 | |
|  | LOCO:FJI | 0.699 | | 0.771 | | 0.446 | | 0.627 | | 555 | |
|  | LOCO:COG | 0.674 | | 0.905 | | 0.522 | | 0.537 | | 6797 | |
|  | LOCO:IRQ | 0.692 | | 0.643 | | 0.378 | | 0.653 | | 9142 | |
|  | LOCO:PAN | 0.751 | | 0.734 | | 0.665 | | 0.704 | | 3659 | |
|  | LOCO:CHN | 0.625 | | 0.466 | | 0.502 | | 0.677 | | 2353 | |
|  | LOCO:PHL | 0.714 | | 0.862 | | 0.65 | | 0.64 | | 18389 | |
|  | LOCO:IDN | 0.596 | | 0.678 | | 0.517 | | 0.544 | | 29390 | |
|  | LOCO:KAZ | 0.637 | | 0.804 | | 0.252 | | 0.472 | | 5891 | |
|  | LOCO:LCA | 0.551 | | 0.641 | | 0.094 | | 0.502 | | 39 | |
|  | LOCO:ZWE | 0.705 | | 0.86 | | 0.75 | | 0.7 | | 33947 | |
|  | LOCO:MLI | 0.694 | | 0.778 | | 0.641 | | 0.63 | | 27350 | |
|  | LOCO:KEN | 0.649 | | 0.793 | | 0.69 | | 0.632 | | 48207 | |
|  | LOCO:MDG | 0.61 | | 0.826 | | 0.714 | | 0.619 | | 37037 | |
|  | LOCO:TUN | 0.763 | | 0.806 | | 0.455 | | 0.652 | | 4748 | |
|  | LOCO:BEN | 0.673 | | 0.793 | | 0.577 | | 0.591 | | 21929 | |
|  | LOCO:SDN | 0.583 | | 0.813 | | 0.442 | | 0.448 | | 7859 | |
|  | LOCO:UZB | 0.662 | | 0.819 | | 0.603 | | 0.599 | | 4838 | |
|  | LOCO:ARM | 0.804 | | 0.81 | | 0.474 | | 0.705 | | 1301 | |
|  | **LOCO-CV mean** | **0.665** | | **0.775** | | **0.533** | | **0.603** | | **285478** | |
| Swine | LOCO:HTI | | 0.565 | | 0.757 | | 0.514 | | 0.518 | | 2832 |
|  | LOCO:NER | | NA | | NA | | NA | | NA | | 0 |
|  | LOCO:FJI | | 0.682 | | 0.868 | | 0.204 | | 0.478 | | 219 |
|  | LOCO:COG | | 0.58 | | 0.924 | | 0.072 | | 0.287 | | 725 |
|  | LOCO:IRQ | | 0.707 | | 0.8 | | 0.003 | | 0.554 | | 15 |
|  | LOCO:PAN | | 0.712 | | 0.83 | | 0.255 | | 0.558 | | 833 |
|  | LOCO:CHN | | 0.687 | | 0.704 | | 0.441 | | 0.622 | | 1424 |
|  | LOCO:PHL | | 0.704 | | 0.855 | | 0.332 | | 0.52 | | 6622 |
|  | LOCO:IDN | | 0.742 | | 0.718 | | 0.178 | | 0.692 | | 3800 |
|  | LOCO:KAZ | | 0.643 | | 0.475 | | 0.081 | | 0.745 | | 1266 |
|  | LOCO:LCA | | 0.686 | | 0.652 | | 0.123 | | 0.778 | | 23 |
|  | LOCO:ZWE | | 0.608 | | 0.828 | | 0.069 | | 0.347 | | 1594 |
|  | LOCO:MLI | | 0.609 | | 0.686 | | 0.059 | | 0.532 | | 961 |
|  | LOCO:KEN | | 0.596 | | 0.167 | | 0.014 | | 0.574 | | 144 |
|  | LOCO:MDG | | 0.699 | | 0.65 | | 0.387 | | 0.663 | | 8775 |
|  | LOCO:TUN | | NA | | NA | | NA | | NA | | 0 |
|  | LOCO:BEN | | 0.614 | | 0.848 | | 0.167 | | 0.381 | | 3283 |
|  | LOCO:SDN | | 0.634 | | 0.494 | | 0.298 | | 0.693 | | 3837 |
|  | LOCO:UZB | | 0.63 | | 0.167 | | 0.001 | | 0.438 | | 6 |
|  | LOCO:ARM | | 0.76 | | 0.754 | | 0.181 | | 0.728 | | 317 |
|  | **LOCO-CV mean** | | **0.659** | | **0.676** | | **0.188** | | **0.562** | | **36676** |
| Ruminants | LOCO:HTI | 0.642 | | 0.815 | | 0.644 | | 0.6 | | 13817 | |
|  | LOCO:NER | 0.686 | | 0.877 | | 0.807 | | 0.727 | | 10006 | |
|  | LOCO:FJI | 0.699 | | 0.866 | | 0.502 | | 0.605 | | 655 | |
|  | LOCO:COG | 0.647 | | 0.802 | | 0.216 | | 0.496 | | 2113 | |
|  | LOCO:IRQ | 0.727 | | 0.703 | | 0.407 | | 0.668 | | 9043 | |
|  | LOCO:PAN | 0.651 | | 0.802 | | 0.236 | | 0.496 | | 887 | |
|  | LOCO:CHN | 0.599 | | 0.631 | | 0.309 | | 0.557 | | 1059 | |
|  | LOCO:PHL | 0.699 | | 0.826 | | 0.331 | | 0.535 | | 6608 | |
|  | LOCO:IDN | 0.647 | | 0.607 | | 0.257 | | 0.601 | | 9293 | |
|  | LOCO:KAZ | 0.708 | | 0.74 | | 0.367 | | 0.595 | | 8427 | |
|  | LOCO:LCA | 0.61 | | 0.824 | | 0.128 | | 0.405 | | 51 | |
|  | LOCO:ZWE | 0.681 | | 0.802 | | 0.689 | | 0.65 | | 31440 | |
|  | LOCO:MLI | 0.703 | | 0.932 | | 0.808 | | 0.738 | | 38159 | |
|  | LOCO:KEN | 0.651 | | 0.762 | | 0.678 | | 0.616 | | 49444 | |
|  | LOCO:MDG | 0.639 | | 0.796 | | 0.547 | | 0.561 | | 21387 | |
|  | LOCO:TUN | 0.772 | | 0.805 | | 0.49 | | 0.674 | | 5140 | |
|  | LOCO:BEN | 0.724 | | 0.715 | | 0.539 | | 0.634 | | 18646 | |
|  | LOCO:SDN | 0.618 | | 0.823 | | 0.624 | | 0.566 | | 12753 | |
|  | LOCO:UZB | 0.749 | | 0.877 | | 0.717 | | 0.681 | | 6000 | |
|  | LOCO:ARM | 0.832 | | 0.743 | | 0.447 | | 0.791 | | 904 | |
|  | **LOCO-CV mean** | **0.684** | | **0.787** | | **0.487** | | **0.61** | | **245832** | |

*Note: LOCO-CV = Leave-One-Country-Out cross-validation. AUC = Area under the receiver operating characteristic curve; F1 = F1-score; n_pos = number of positive (ownership) observations; n_obs = total number of observations in held-out country. The bottom row of each model section shows the mean metrics across all held-out countries (fixed effects only)*

| Table S3: Variance-Inflation Factors (VIF) by Livestock Component | | | |
| --- | --- | --- | --- |
| Covariate | **Poultry VIF** | **Swine VIF** | **Ruminants VIF** |
| time | 1.030 | 1.078 | 1.033 |
| accessibility | 1.311 | 1.266 | 1.328 |
| aridity | 23.417 | 23.234 | 24.662 |
| cropland | 1.783 | 1.520 | 1.912 |
| density | 1.777 | 1.620 | 1.735 |
| elevation | 2.572 | 3.232 | 2.582 |
| evi | 2.539 | 2.538 | 2.462 |
| footprint | 2.382 | 1.991 | 2.358 |
| gdp | 1.865 | 1.855 | 1.935 |
| gini | 1.118 | 1.194 | 1.113 |
| growing | 4.701 | 3.596 | 4.715 |
| hdi | 1.893 | 1.995 | 1.925 |
| irrigation | 2.034 | 1.680 | 2.243 |
| lst_range | 4.799 | 4.699 | 4.584 |
| moisture | 3.312 | 2.965 | 3.320 |
| night_light | 2.723 | 2.392 | 2.609 |
| pasture | 1.419 | 1.438 | 1.447 |
| pet | 3.707 | 3.315 | 3.677 |
| precipitation | 21.935 | 22.082 | 23.180 |
| water_dist | 1.066 | 1.052 | 1.063 |
| climate | 4.863 | 4.519 | 4.916 |
| land | 4.352 | 3.899 | 4.085 |
| soil | 1.458 | 1.348 | 1.451 |
| urban | 2.770 | 2.533 | 2.457 |

*Variance-inflation factors quantify covariate collinearity for each livestock taxon model. Values <5 indicate low collinearity; values 5–10 indicate moderate collinearity; values >10 indicate high collinearity. Development-related covariates (nighttime lights, built-up footprint, population density) show moderate collinearity (VIF 3–8), reflecting spatial co-occurrence in urbanized areas. This collinearity does not bias predictions but may affect interpretability of individual SHAP values in correlated variable clusters.*

| Table S4: Raster Covariate Missingness | | |
| --- | --- | --- |
| Covariate | **N Missing Pixels** | **Percent Missing (%)** |
| accessibility | 2,216 | 0.079 |
| aridity | 2,185 | 0.078 |
| climate | 2,185 | 0.078 |
| cropland | 2,185 | 0.078 |
| density | 2,185 | 0.078 |
| elevation | 2,185 | 0.078 |
| evi | 2,293 | 0.082 |
| footprint | 4,724 | 0.168 |
| gdp | 2,257 | 0.080 |
| gini | 2,248 | 0.080 |
| growing | 2,191 | 0.078 |
| hdi | 2,341 | 0.083 |
| irrigation | 0 | 0.000 |
| land | 5,032 | 0.179 |
| lst_range | 2,185 | 0.078 |
| moisture | 2,748 | 0.098 |
| night_light | 2,185 | 0.078 |
| pasture | 2,185 | 0.078 |
| pet | 2,394 | 0.085 |
| precipitation | 9,625 | 0.343 |
| soil | 2,347 | 0.084 |
| urban | 2,185 | 0.078 |
| water_dist | 2,193 | 0.078 |

*Per-covariate missing pixel counts and percentages on land cells within the prediction domain. Most covariates have <1% missing pixels. Missing pixels were imputed using k-nearest neighbours (k=10) prior to model fitting, preserving spatial structure while assuming local covariate homogeneity. Predictions in regions with high covariate missingness may have elevated uncertainty.*

## ***Guideline compliance***

| Table S5: Checklist for Guidelines for Accurate and Transparent Health Estimates Reporting (GATHER) (3) | |
| --- | --- |
| Objectives and funding | |
| 1. Define the indicator(s), populations (including age, sex, and geographic entities), and time period(s) for which estimates were made. | Methods - objectives and scope section |
| 1. List the funding sources for the work | Funding sources are listed in the financial support section |
| Data inputs | |
| 1. Describe how the data were identified and how the data were accessed. | Methods section and table 2 |
| 1. Specify the inclusion and exclusion criteria. Identify all ad-hoc exclusions. | Methods section |
| 1. Provide information on all included data sources and their main characteristics. For each data source used, report reference information or contact name/institution, population represented, data collection method, year(s) of data collection, sex and age range, diagnostic criteria or measurement method, and sample size, as relevant. | Table 2 and Supplementary File S2 |
| 1. Identify and describe any categories of input data that have potentially important biases (e.g., based on characteristics listed in item 5). | Not applicable |
| 1. Describe and give sources for any other data inputs. | Methods section |
| 1. Provide all data inputs in a file format from which data can be efficiently extracted (e.g., a spreadsheet rather than a PDF), including all relevant meta-data listed in item 5. For any data inputs that cannot be shared because of ethical or legal reasons, such as third-party ownership, provide a contact name or the name of the institution that retains the right to the data. | Not applicable, see data availability statement. |
| Data analysis | |
| 1. Provide a conceptual overview of the data analysis method. A diagram may be helpful. | Methods –analysis section |
| 1. Provide a detailed description of all steps of the analysis, including mathematical formulae. This description should cover, as relevant, data cleaning, data pre-processing, data adjustments and weighting of data sources, and mathematical or statistical model(s). | Methods –analysis section; Supplementary File S1 |
| 1. Describe how candidate models were evaluated and how the final model(s) were selected. | Methods – analysis section |
| 1. Provide the results of an evaluation of model performance, if done, as well as the results of any relevant sensitivity analysis. | Figure 3 |
| 1. Describe methods of calculating uncertainty of the estimates. State which sources of uncertainty were, and were not, accounted for in the uncertainty analysis. | Methods – analysis section |
| 1. State how analytical or statistical source code used to generate estimates can be accessed. | See data availability statement. The statistical source code used to generate estimates can be accessed is available from the corresponding author upon reasonable request. |
| Results and discussion | |
| 1. Provide published estimates in a file format from which data can be efficiently extracted. | Supplementary TIFF files of estimates. Supplementary files 3a-c |
| 1. Report a quantitative measure of the uncertainty of the estimates (e.g., uncertainty intervals). | Additional TIFF files giving standard errors of the estimates are provided as Supplementary files 3d-f |
| 1. Interpret results in light of existing evidence. If updating a previous set of estimates, describe the reasons for changes in estimates. | Discussion section |
| 1. Discuss limitations of the estimates. Include a discussion of any modelling assumptions or data limitations that affect interpretation of the estimates. | Discussion section |

## **References**

1. Cameletti M, Lindgren F, Simpson D, Rue H. Spatio-temporal modeling of particulate matter concentration through the SPDE approach. AStA Adv Stat Anal. 2013 Apr 1;97(2):109–31. doi:10.1007/s10182-012-0196-3

2. Simpson D, Rue H, Riebler A, Martins TG, Sørbye SH. Penalising Model Component Complexity: A Principled, Practical Approach to Constructing Priors. Statistical Science. 2017 Feb;32(1):1–28. doi:10.1214/16-STS576

3. Stevens GA, Alkema L, Black RE, Boerma JT, Collins GS, Ezzati M, et al. Guidelines for Accurate and Transparent Health Estimates Reporting: the GATHER statement. The Lancet. 2016 Dec 10;388(10062):e19–23. doi:10.1016/S0140-6736(16)30388-9
