# Supplementary material for: Mapping the prevalence of household-scale livestock ownership by animal taxon in low- and middle-income countries: A prediction model using template model builder
Source: PLoS One. 2026 Jul 31;21(7):e0355207. doi: 10.1371/journal.pone.0355207 (PMC13426965; doi:10.1371/journal.pone.0355207)
Supplement: Attachment — Submitted filename: PONE-D-26-09292 Response to review 1.docx [file pone.0355207.s005.docx]

# PONE-D-26-09292 Response to Review 1

*We thank the editor and reviewer for their helpful reviews. We have addressed each comment below with our responses in italics. As requested, we have provided both a marked-up copy of the amended manuscript as well as an unmarked version with the changes incorporated. Responding to this review has significantly improved the manuscript and we ask you to again consider this for publication in PLOS One. Please note that we have changed the analysis significantly since the initial submission, partly in response to the review. The following changes have been made:*

a. *We have replaced the INLA modeling approach with the more computationally efficient RTMB R package. This has allowed us to fit the model to the whole database, negating the need to sub-sample one household per cluster.*
b. *We have updated the input database with new surveys that have been released since we conducted the initial analysis.*
c. *We have added three new covariates. We have also removed the region covariate that we had previously included, since this was producing sharp border effects to the predictions along the region boundaries. The exception to this is the swine model, for which we allowed some of the aspects of the model to vary by region, as outlined below and in the methods section.*

## Editor’s comments:

1. Please ensure that your manuscript meets PLOS ONE's style requirements, including those for file naming.

*We have amended the manuscript to meet the guidelines.*

2. Please note that PLOS One has specific guidelines on code sharing for submissions in which author-generated code underpins the findings in the manuscript. In these cases, we expect all author-generated code to be made available without restrictions upon publication of the work. Please review our guidelines at <https://journals.plos.org/plosone/s/materials-and-software-sharing#loc-sharing-code> and ensure that your code is shared in a way that follows best practice and facilitates reproducibility and reuse.

*We have made the code available in this repository* [*https://doi.org/10.18130/V3/B9Z4QA*](https://doi.org/10.18130/V3/B9Z4QA)*.*

3. We note that the grant information you provided in the ‘Funding Information’ and ‘Financial Disclosure’ sections do not match. When you resubmit, please ensure that you provide the correct grant numbers for the awards you received for your study in the ‘Funding Information’ section.

*We were unable to locate the “Financial Disclosure” section upon resubmission. The amended statement should read as follows:*

*This research was supported financially by the National Institutes of Health’s National Institute of Allergy and Infectious Diseases (grants 1K01AI168493-01A1 to JMC and 1R03AI151564-01 to MNK) and Fogarty International Center (grant K43TW012298 to FSS); the European Union under the HORIZON EUROPE Programme (Grant Agreement Number: 101137255 with sub-award to JMC); the UVA Environmental Institute through the Strategic Investment Fund (to NH); and the University of Virginia’s Engineering in Medicine (EIM) funding program (to VL). The funders played no role in the design and implementation of the study or the analysis and interpretation of the results.*

4. For studies involving third-party data, we encourage authors to share any data specific to their analyses that they can legally distribute. PLOS recognizes, however, that authors may be using third-party data they do not have the rights to share. When third-party data cannot be publicly shared, authors must provide all information necessary for interested researchers to apply to gain access to the data. (<https://journals.plos.org/plosone/s/data-availability#loc-acceptable-data-access-restrictions>) For any third-party data that the authors cannot legally distribute, they should include the following information in their Data Availability Statement upon submission:
   a. A description of the data set and the third-party source
   b. If applicable, verification of permission to use the data set
   c. Confirmation of whether the authors received any special privileges in accessing the data that other researchers would not have
   d. All necessary contact information others would need to apply to gain access to the data

*See the amended data availability statement which reads:*

*The human subject data used in this analysis were collected and owned by third parties, not by the authors. They are all publicly available and can be accessed from the following sources (also listed in supplementary file S2) in the same manner that the authors accessed them: - Demographic and Health Surveys, Malaria Indicator Surveys, and AIDS Indicator Surveys - https://dhsprogram.com/ - Multiple Indicator Cluster Surveys - China Family Panel Studies - https://opendata.pku.edu.cn/dataverse/CFPS. The data on the covariate predictors are available from the sources cited in Table 2. The authors do not have the right to share these datasets but did not have any special access privileges that others would not have. The prediction datasets resulting from this analysis are publicly available through the Dryad repository at:* [*https://doi.org/10.5061/dryad.n2z34tnb3*](https://doi.org/10.5061/dryad.n2z34tnb3)*. The statistical code used to generate them can be found here* [*https://doi.org/10.18130/V3/B9Z4QA*](https://doi.org/10.18130/V3/B9Z4QA)

5. We note that Figures 1,2 and Figure S2 in your submission contain [map/satellite] images which may be copyrighted. All PLOS content is published under the Creative Commons Attribution License (CC BY 4.0), which means that the manuscript, images, and Supporting Information files will be freely available online, and any third party is permitted to access, download, copy, distribute, and use these materials in any way, even commercially, with proper attribution. For these reasons, we cannot publish previously copyrighted maps or satellite images created using proprietary data, such as Google software (Google Maps, Street View, and Earth). We require you to either (1) present written permission from the copyright holder to publish these figures specifically under the CC BY 4.0 license, or (2) remove the figures from your submission: You may seek permission from the original copyright holder of Figures 1,2 and Figure S2 to publish the content specifically under the CC BY 4.0 license.

*The images in those figures are not copyrighted. We compiled the basemaps using publicly available shapefiles, and we already specified the sources and their lack of restrictions on use in the figure labels but have now added URLs and their CC0 designation.*

6. We are unable to open your Supporting Information file [Livestock_ownership_supplementary_file_3a,3b,3c,3d,3e,3f_Poultry.tif]. Please kindly revise as necessary and re-upload.

*Those files are in a zipped folder that is quite large (>87MBs). We now specify in the data availability statement that those files are available in a Dryad repository at this URL* [*https://doi.org/10.5061/dryad.n2z34tnb3*](https://doi.org/10.5061/dryad.n2z34tnb3)*.*

7. If the reviewer comments include a recommendation to cite specific previously published works, please review and evaluate these publications to determine whether they are relevant and should be cited. There is no requirement to cite these works unless the editor has indicated otherwise.

*The reviewer’s comments did not include any such recommendations.*

## Reviewers' comments:

### Reviewer #1:

- This manuscript uses publicly available household survey microdata from DHS, MICS, and CFPS to model and map the prevalence of household-scale livestock ownership for poultry, swine, and ruminants across low- and middle-income countries using INLA-based spatial logistic models. The topic is important, the dataset is large, and the resulting maps could be useful for zoonotic disease research. The paper is generally well written and potentially suitable for publication, but several aspects of the modelling strategy, validation, assumptions, and interpretation need clarification or strengthening before the conclusions can be fully supported.

*We appreciate the positive feedback and are happy to address the reviewer’s comments.*

#### Major comments:

1. The manuscript notes that the semi-variogram for swine suggests a non-stationary spatial process that may violate the stationary Matérn assumption, yet the same stationary INLA specification is retained for all three taxa. This is a substantive technical concern because swine ownership is also the most culturally constrained and spatially heterogeneous outcome. I suggest the authors either fit a more flexible model for swine, such as region-specific spatial structure or another approach that relaxes stationarity, or provide stronger evidence that the current specification is adequate and then moderate the interpretation of the swine map in the abstract and discussion.

*We agree this was a substantive problem and have implemented a region-specific spatial structure for swine. We implemented a Template Model Builder (RTMB) and now fit swine with region-specific Matérn kernels by including region variable as fixed effects, in response to the non-stationarity evident in the swine semi-variogram. Poultry and ruminants retain a single global SPDE, as their semi-variograms are consistent with stationarity. Figure S1 now displays fitted Matérn curves overlaid on Pearson residual semi-variograms. For swine, region-stratified residuals show substantially tighter clustering around the fitted curves. We have moderated language in the abstract and discussion to describe swine predictions in data-sparse regions as regional patterns constrained by covariate similarity rather than fine-scale measurements.*

2. The manuscript reports ROC-AUC, recall, precision, accuracy, and F1-score, and describes performance as impressive, but it is unclear whether these metrics were obtained in-sample, through random cross-validation, or using spatially separated folds. For spatial prediction models, random validation can give over-optimistic results because nearby observations are not independent. The paper should state exactly how performance was assessed and ideally report spatial cross-validation or another out-of-sample strategy that reflects the intended use of the maps.

*This is an important issue. The original manuscript reported in-sample (training) metrics, which are not informative for spatial prediction. We now include leave-one-country-out (LOCO) spatial cross-validation as the primary performance metric. Under LOCO-CV, all observations from a single country are excluded, the models are refitted to the remaining data, predictions are made for left-out households, and performance metrics (recall, precision, accuracy, F1-score, and area under the receiver operating characteristic curve (ROC-AUC) statistics) are computed per fold and averaged. To manage computational cost, we evaluated a stratified random subsample of 20 countries balanced across region variable and livestock components. Both in-sample and LOCO-CV metrics are now provided in Table S2 and discussed in the Discussion section.*

3. According to the Methods, one household with non-missing outcome per cluster was retained for analysis to reduce database size. This choice discards substantial within-cluster information and may affect both prevalence estimation and covariate importance. At minimum, the authors should explain the implications more clearly.

*We agree and have removed the within-cluster sub-sampling entirely. The original INLA framework took one household per cluster (~235,000 observations) for computation. The transition to RTMB allows us to retain all ~5.0–5.3 million households while modeling within-cluster correlation via a cluster-level random intercept. We validated the revised full-data RTMB results (Supplementary Table S2). These differences reflect improved use of within-cluster information and are documented in the Methods and Discussion.*

4. In majority-Muslim countries where swine ownership was not asked in any survey, households were coded as ‘no’ rather than missing. Although this may often be reasonable, it is still an assumption that may not hold and could influence both model fit and predicted prevalence in sparse-data regions. I suggest the authors run the analysis with these data as missing to see if the results are significantly altered. Reporting these findings would provide a fuller justification and discussion of the possible bias and would strengthen the manuscript.

*We agree and have conducted this sensitivity analysis. The preprocessing pipeline was conducted by switching between two coding schemes: "zero" (baseline, households coded as not owning swine) and "missing" (sensitivity analysis, households dropped entirely). We refit the swine model under both schemes. Results are robust: the predicted band of higher swine ownership across central-southern China, southeastern Asia, and northeast India is reproduced in both coding schemes. The most visible differences occur in Middle East and North Africa and parts of Sub-Saharan Africa with high Muslim fractions, where the "missing" scheme produces marginally higher predicted swine probability (typically <0.05) due to increased reliance on covariate extrapolation.*

5. The Results acknowledge major geographic gaps, including many South American countries and several countries without public surveys or without livestock questions. In those settings, the model is extrapolating largely from covariates and broad regional structure, which likely increases uncertainty. I suggest distinguishing more explicitly between data-rich and data-poor regions in the Discussion and include clearer communication of predictive uncertainty rather than focusing mainly on average predicted prevalence.

*We acknowledge this in the limitations paragraph and have now further elaborated on it.*

6. The manuscript occasionally overstates what the maps represent. The study estimates binary household ownership of broad livestock taxa, not animal density, husbandry intensity, within-household contact, or disease transmission risk itself. For that reason, statements suggesting that the maps identify areas of high exposure to animal disease reservoirs should be softened. These outputs are better described as geographically resolved proxy exposure layers that may support future zoonotic risk analyses when combined with other epidemiologic or environmental data.

*We have toned down this language in the relevant places.*

*Minor comments:*

7. The manuscript would benefit from a brief discussion of covariate collinearity. Several predictors appear likely to be correlated, including built-up area, nighttime lights, and other development-related measures. Because SHAP values are presented as indicators of variable contribution, the paper should note that interpretation of individual covariates may be unstable when predictors are strongly correlated.

*We assessed covariate collinearity using variance-inflation factors (VIF) computed on the fitted logistic models. VIF values are reported in Supplementary Table. Development-related covariates (nighttime lights, built-up footprint, population density) show moderate collinearity (VIF 3–8), reflecting spatial co-occurrence of urban infrastructure. Importantly, this collinearity does not bias predictions. However, it does affect interpretability of individual covariate effects. We now clarify in the Methods that SHAP values represent marginal contributions conditional on all other predictors in the model. In clusters of correlated variables, individual SHAP rankings may reflect collinearity noise rather than true importance differences. Readers should interpret covariate contributions holistically rather than assigning independent significance to any single development metric.*

8. Missing raster pixels were filled using k-nearest neighbours, but no details are given about the extent of missingness or the likely effect on prediction uncertainty. A short explanation in the Methods and a brief note in the limitations would help.

*Per-covariate raster missingness is quantified and reported in Supplementary Table S6. Across the prediction grid, most covariates had <2% missing pixels on land. Missing raster pixels were filled using k-nearest neighbours imputation (k=10, R gstat package) prior to prediction. This approach preserves spatial structure but assumes local covariate homogeneity. We note in the Limitations that predictions in regions with high raster missingness should be interpreted with additional caution, as KNN-imputed values may not fully capture true local covariate variation.*
